# Whole-Genome Synthesis and Characterization of Viable S13-Like Bacteriophages

**DOI:** 10.1371/journal.pone.0041124

**Published:** 2012-07-18

**Authors:** Yuchen Liu, Yonghua Han, Weiren Huang, Yonggang Duan, Lisha Mou, Zhimao Jiang, Pingping Fa, Jun Xie, Ruiying Diao, Yuanbin Chen, Yiwang Ye, Ruilin Yang, Jing Chen, Xiaojuan Sun, Zesong Li, Aifa Tang, Yaoting Gui, Zhiming Cai

**Affiliations:** 1 Guangdong and Shenzhen Key Laboratory of Male Reproductive Medicine and Genetics, Institute of Urology, Peking University Shenzhen Hospital, Shenzhen PKU-HKUST Medical Center, Shenzhen, China; 2 Shantou University Medical College, Shantou, China; 3 The Institute of Urogenital Diseases, Shenzhen University, Shenzhen, China; 4 Shenzhen Second People's Hospital, Shenzhen, China; Virginia Tech, United States of America

## Abstract

**Background:**

Unprecedented progresses in high-throughput DNA sequencing and de novo gene synthesis technologies have allowed us to create living organisms in the absence of natural template.

**Methodology/Principal Findings:**

The sequence of wild-type S13 phage genome was downloaded from GenBank. Two synonymous mutations were introduced into wt-S13 genome to generate m1-S13 genome. Another mutant, m2-S13 genome, was obtained by engineering two nonsynonymous mutations in the capsid protein coding region of wt-S13 genome. A chimeric phage genome was designed by replacing the F capsid protein open reading frame (ORF) from phage S13 with the F capsid protein ORF from phage G4. The whole genomes of all four phages were assembled from a series of chemically synthesized short overlapping oligonucleotides. The linear synthesized genomes were circularized and electroporated into E.coli C, the standard laboratory host of S13 phage. All four phages were recovered and plaques were visualized. The results of sequencing showed the accuracy of these synthetic genomes. The synthetic phages were capable of lysing their bacterial host and tolerating general environmental conditions. While no phenotypic differences among the variant strains were observed when grown in LB medium with CaCl_2_, the S13/G4 chimera was found to be much more sensitive to the absence of calcium and to have a lower adsorption rate under calcium free condition.

**Conclusions/Significance:**

The bacteriophage S13 and its variants can be chemically synthesized. The major capsid gene of phage G4 is functional in the phage S13 life cycle. These results support an evolutional hypothesis which has been proposed that a homologous recombination event involving gene F of quite divergent ancestral lineages should be included in the history of the microvirid family.

## Introduction

The successful development of the high-throughput DNA sequencing technology opened the door for a quantum leap in science advancement [1]. As genomes of many new species are being sequenced almost daily, genomic data are rapidly accumulating. Thanks to such sequence information, synthetic biologists are attempting to create novel living systems [2]–[3]. Rapid progress in DNA synthesis has been extended to the level of organism whole-genome de novo synthesis in the absence of a natural template [4]–[5]. In 2002, work in Wimmer's group led to the first chemical synthesis of a DNA corresponding to the whole genome of poliovirus [6]. In 2003, Smith et al. [7] described a stepwise synthesis of the genome of bacteriophage ΦX174 just in two weeks. In 2005, Endy and colleagues [8] reconstructed the genome of bacteriophage T7 by removing complex gene overlaps. In 2010, Gibson et al. [9] achieved the first construction of a self-replicating bacterial cell controlled only by a chemically synthesized genome.

Bacteriophage S13, a small phage active against *Escherichia coli*, is one of a series of virulent phages originally isolated from *Salmonella typhimurium* [10]. It is a member of the *Microviridae*. The nucleotide sequence and genome organization of bacteriophage S13 DNA were determined in 1985 [11] and again in 2000 [12]. The phage has a small circular single-stranded DNA that replicates through a double-stranded replicative form of DNA. It has been shown to be closely related to bacteriophage ΦX 174. Genomes of the two phages are 5386 bp long and differ at about 111 nucleotide sites [11]. G4 is also treated as a S13-like phage and its genome is 191 nucleotides longer than the S13 genome [13]–[14]. Their genomes code for 11 proteins, including A, A*, B, C, D, E, F, G, H, J and K. Among these proteins, A, A*, B, C and D are involved in phage DNA replication and assembly, F, G, H and J are phage structural proteins, E is a lysis protein and K may be beneficial for the growth of the phage [15].

Horizontal gene transfer and recombination have long been hypothesized to play an important role in the evolutionary history of the microvirid family [16]. In terms of horizontal evolution, it is important to know how much the genome can change during homologous recombination and still encode a viable phage [17]. The effects of genetic recombination and complementation on phage viability were evaluated to elucidate the evolutionary interrelationship between the three related phages [18]–[22].

In this paper, we describe the chemical synthesis of S13-like bacteriophages in the test tubes and characterize their biological properties. The synthetic phages can be used as a tool for better understanding the commonalities of related phage genes and the theory of horizontal evolution.

## Results

### Chemical synthesis of the genomes of all four bacteriophages

**Figure 1:**
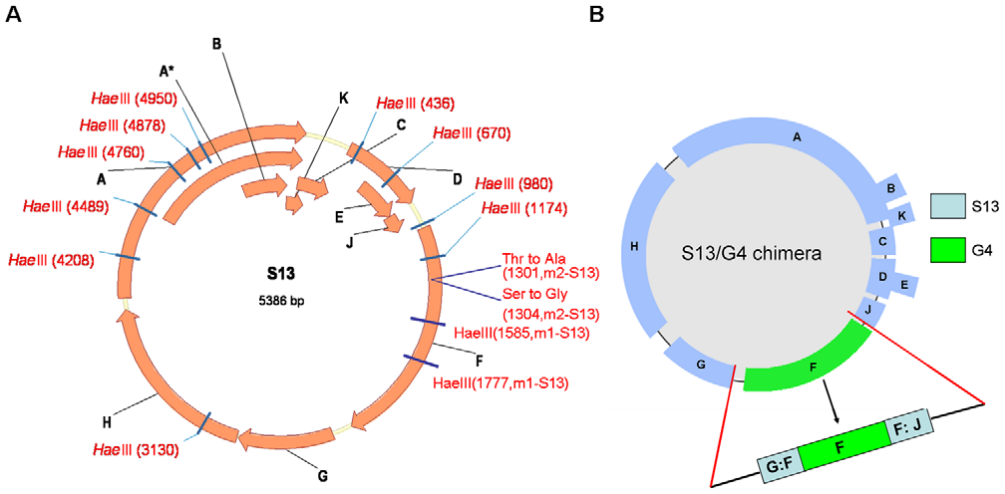
Genomic structures of our synthetic bacteriophages. (A) The S13 genome is 5386 nucleotides long and it codes for 11 genes. The four mutation sites are located at the F coding region. (B) S13/G4 chimeric recombinant genome. This new phage genotype consists of the S13 genome with its allele of F replaced by the homologue from G4. Blue areas represent regions of S13 phage and the green area represents the F gene of G4 phage. The label “G:F” represents the untranslated region between genes G and F and the other label “F:J” represents the untranslated region between genes F and J.

The oligonucleotides were designed to synthesize the wild type S13 genome with exactly the same sequence reported by H. A. Wichman et al. in 2000 (GenBank accession number AF274751.1). Two of the same restriction endonuclease sites(*HaeIII*) and two nonsynonymous mutation sites within the F coding region were introduced into the wild-type sequence respectively to create m1-S13 and m2-S13 genomes (Fig. 1A). To investigate whether the major capsid gene of G4 could functionally interact with other protein coding genes of S13 during the complete phage life cycle, we constructed a recombinant chimeric phage consisting of the A, A*, B, C, D, E, G, H, J and K genes from S13 and the F gene from G4 (Fig. 1B). The full-sized genomes of S13 DNA molecules were completed through a series of steps as described in materials and methods. A more detailed report was shown in Material S1.

### Circularization of the linear full-length DNAs and recovery of infectious phage particles

**Figure 2:**
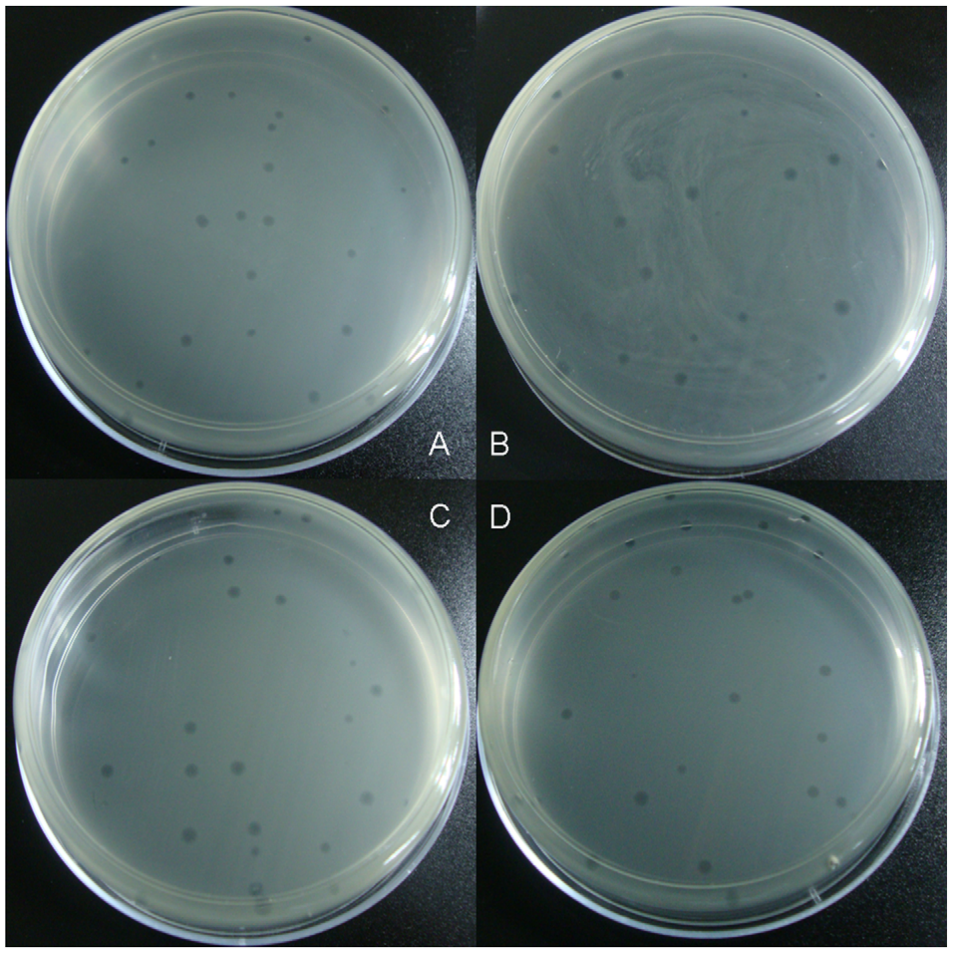
Images of the plaques (8 h, 37°C, 10 cm Petri dish). A. wt-S13 B. m1-S13 C. m2-S13 D. S13/G4 chimera.

Because of S13 sequence restoring a *PstI* site at each end, the PCR productions of full-length genomes were circularized by ligation with T4 ligase using recommended conditions. To assay the infectivity of circular molecules, each ligation product was electroporated into *E.coli* C cells, which was the standard laboratory host of S13 phage. Plates were incubated overnight at 37°C and phage plaques were visualized after that. Plaques of the synthetic phages on *E. coli* strain C on agar plate were 1 or 2 mm in diameter. They formed small, clear, round plaques and some merge together to form a mass (Fig. 2).

### Restriction digestion of bacteriophage S13 DNA and detection of engineered genetic markers

**Figure 3:**
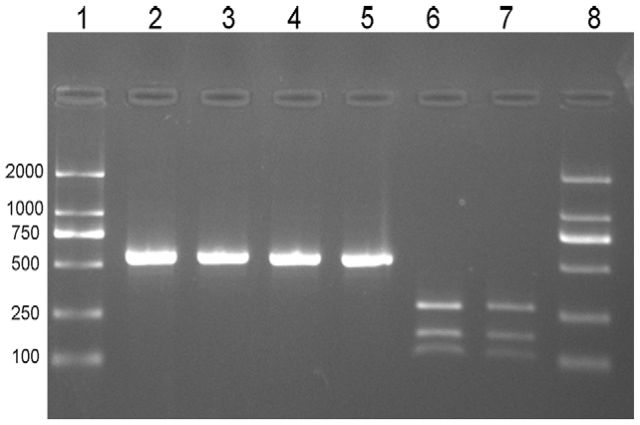
Test for the presence of engineered *HaeIII* genetic markers in m1-S13. Lane 1 and lane 8 contained the D2000marker. As expected, only a single band of 628 bp was observed when PCR products of wt-S13 were digested with *HaeIII*, due the absence of the genetic marker (lane 2 and 3). A single band of 628 bp was observed when PCR products of m1-S13 were not digested with *HaeIII* (lane 4 and 5). Electrophoretic analysis of the PCR products of the m1-S13 showed the presence of 3 bands (316nt,192 nt and 120nt) after digestion with *HaeIII*, an observation indicating that the synthetic phage contained the engineered markers(lane 6 and 7). The values to the left are molecular sizes in base pairs (bp).

DNA of both wt-S13 and m1-S13 phages were successfully isolated and purified from plaques. The specific 628 nt PCR fragments were amplified using the specific primer pair. The resulting PCR products were either treated or untreated with restriction endonuclease *HaeIII* and subsequently subjected to electrophoretic analyses. As shown in Fig. 3, m1-S13 could be digested, but wt-S13 was not sensitive to *HaeIII.* The different banding patterns confirmed that the genetic markers were existed in our synthetic phage DNA.

### Sequencing results of all four bacteriophages

To further confirm that the DNA of each phage was the designed synthetic DNA sequence, we picked one large plaque from each plate and sequenced many important regions of the genome. The phage from each resulting plaque was verified to be the corresponding synthetic strain (data shown in File . The sequencing results also showed the accuracy of these synthetic genomes.

### Phylogenetic tree

**Figure 4:**
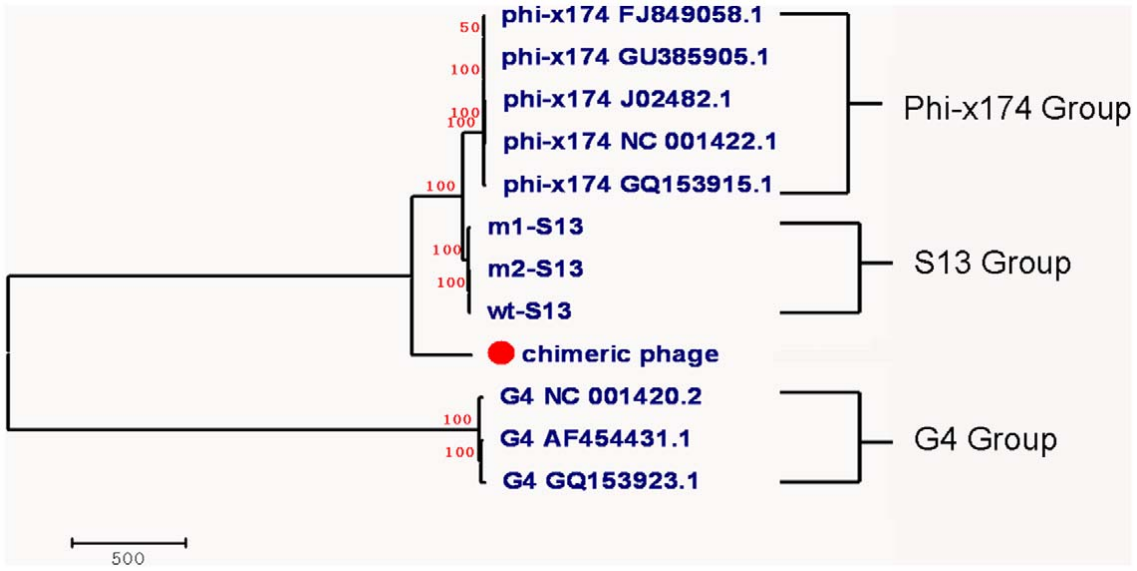
A phylogenetic tree constructed by using the maximum parsimony method. Sequence cluster analysis based on the whole-genome sequences of our synthetic phages and other related phage sequences obtained from the GenBank database.

To analyze the DNA sequence clusters between the synthetic S13-like phages and eight other previously sequenced microvirid phages, we constructed of a phylogenetic tree by using maximum parsimony method with MEGA v 5.0 software(Fig. 4). As shown in this tree, the phages could be divided into 4 clades with distinct local characteristics. Phage wt-S13, m1-S13 and m2-S13 formed the S13 group, which was a close relative of the ΦX174 group. The chimeric phage might belong to another clade of the *Microviridae*, which was very different from the ΦX174 or G4 group.

### Assessment of phage lysis efficiency

**Figure 5:**
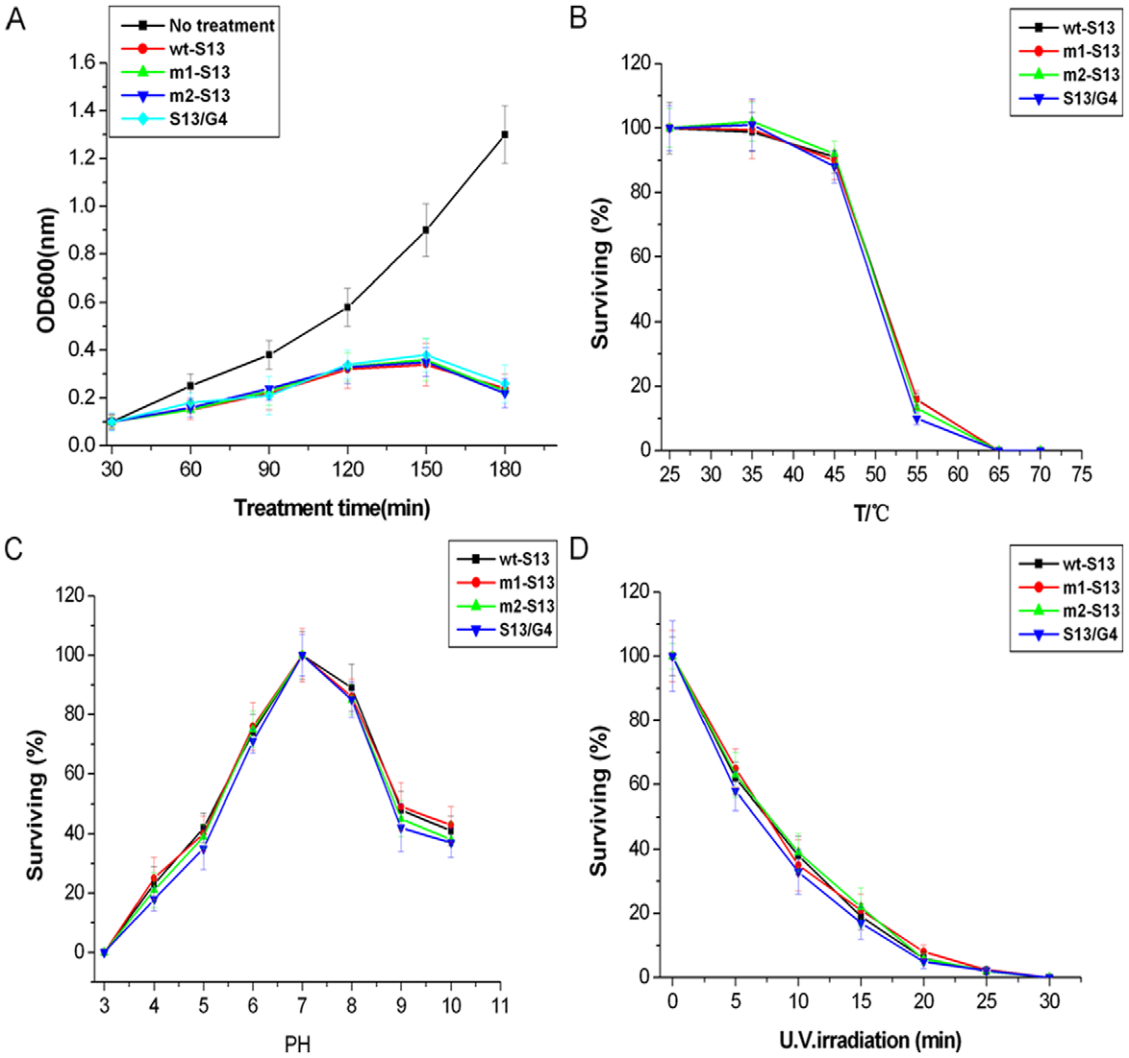
Biological characteristics of the synthetic phages. (A)Time courses of host cell lysis by synthetic phages. A growth curve for *E. coli* C with no treatment was reproduced for comparison. (B)Temperature stability test. Phages were incubated under different temperature values for 30 min before determining the percentage of surviving infectious phages. (C) pH stability test. Phages were incubated under different pH values for 30 min before determining the percentage of surviving infectious phages. (D) Inactivation of phages by ultraviolet irradiation. Samples were taken at different time intervals to calculate the percentage of surviving infectious phages. Results were shown in Mean ± S.D. from three independent experiments.

Phage samples were prepared at intervals of 30 minutes to measure the changes in OD600nm. The antibacterial effects of synthetic phages on *E.coli* strain C (at an MOI of 1) were shown in Fig. 5A. 60 minutes after the time of infection, the prominent suppressive effects of the phages on bacterial growth were observed. The OD600 values started to decrease gradually at 150 minutes after phages infection. Taken together, we found no obvious differences in the killing abilities of the four phages (p>0.05). A growth curve for the same strain with no treatment was shown for comparison. The result demonstrated that OD600 kept increasing when the *E.coli* strain C was not infected.

### Effects of temperature, pH and ultraviolet radiation

Thermal stability test was carried out to analyze the heat resistant capability of each type of phage. All phage particles were not affected by a 30 minutes exposure to a temperature of 25 or 35°C in LB broth, whereas some inactivation was observed at 45 and 55°C. No survivors could be recovered after a 30 minutes exposure to 65 or 70°C (Fig. 5B). The results showed that synthetic phages were heat stable at normal temperatures.

An exposure of 30 minutes in LB broth previously adjusted to pH 3 inactivated all phage particles. At PH 4, 5, 6, 8, 9 and 10, some of the phage particles survived. Maximum survivals were at pH 7 (Fig. 5C). These results suggested that extreme pHs could affect synthetic phages stabilities.

Partial inactivation of all four phages occurred exponentially after 5 minutes of irradiation. The inactivation curves for these phages by ultraviolet radiation were illustrated in Fig. 5D.

These results together indicated that synthetic phages had high degrees of stability in different environments. No great differences were observed among these four phages (p>0.05).

### Adsorption rates assay

The adsorption rates for the wt-S13 and each mutant were determined by performing an adsorption rate assay. This result was consistent across the four different phages when the LB medium was supplemented with CaCl_2_ (data not shown). We then re-estimated the values from this study in LB medium without CaCl_2_. Adsorption rate was approximately three times greater for each S13 phage than for the S13/G4 chimera (p<0.05) (Table 1). The differences were large enough to suggest that the chimera was far more sensitive to low levels of calcium.

**Table 1:** Adsorption rates of the synthetic phages.

Viral strain	Adsorption rate (×10^−9^ml/min) a
wt-S13	5.8±0.15
m1-S13	5.7±0.12
m2-S13	5.5±0.18
S13/G4	2.0±0.11*

a The values are mean ± standard errors. *p<0.05 compared with other S13 phages.

## Discussion

To the best of our knowledge, this is the first synthesis of a mosaic phage genome and only the second synthesis of a ΦX174-like phage. Our work demonstrates that bacteriophage S13 can be resurrected from pure genomic information. We provide proof that the genome sequence originally reported by H. A. Wichman et al. [12] in 2000 is accurate. We also confirm the presence or absence of the additional markers in m1-S13 and wt-S13. Both of the amino acid changes in the major capsid protein of m2-S13 have been shown to have no significant influence on phage replication and growth in *E. coli* C culture. As a matter of fact, computational comparisons of the sequences available in GenBank show that these alterations are ubiquitous to both S13 and ΦX 174 genomes.

The major capsid protein gene (F) is the most conserved gene among S13 and its related phages. A recombination event involving gene F of quite divergent genotypes may have occurred in the history of microvirid phages [16]. It has been concluded in the previous work that G4 F protein can function in the ΦX174 infective cycle [21]. Comparison of the nucleotide sequence of S13 with that of G4 makes it clear that the whole genome organization of S13 is the same as that of G4. The protein F of S13 has an average of 69% amino acid sequence similarity compared to that of G4. Based on these findings, we assume that the gene F from S13 can be functionally replaced by the homologue from G4.

Using S13/G4 chimeric genomic DNA as a genetic tool, we have investigated the effect on the phage viability of exchanging the major capsid gene between two different phage types. Our results demonstrate that the F gene of G4 can interact with the non-coat genes of S13 to efficiently reproduce the complete phage life cycle, including the production of viable phage particles. Various antimicrobial efficiencies and environmental stabilities of the chimeric phages are similar to that of wild type S13. From these results, we infer that the species barrier between S13 and G4 is weak. We also believe that the evolutionary hypothesis first proposed by Rokyta et al. [16] to reveal the horizontal transfer of gene F is correct.

In the earlier studies, calcium is known to strongly affect adsorption rates for many phages [23]–[24]. G4 phage has been shown to be sensitive to the absence of calcium [25]. The lower adsorption rate of S13/G4 chimera under calcium free condition is not unexpected because adsorption is thought to be controlled by F protein [26]. So we can change the adsorption rate of a microvirid phage by exchanging the F capsid protein ORF.

It is surprising that there seems to be no strong conflict between the exchanged coat protein (F) and the two capsid proteins G and J. One possible explanation is that the amino acid residues involved in the interactions are highly conserved throughout evolution [27]–[28].

Chemical synthesis of phage genomes in the absence of natural template provides a new and powerful tool for studying phage genetics. Further researches are still needed to explain our consequences.

## Materials and Methods

### Strains and growth media

*Escherichia coli* C, the standard laboratory host, was purchased from the American Type Culture Collection (ATCC no.13706). Cells were cultured in LB medium (10 g NaCl, 10 g tryptone, and 5 g yeast extract per liter) supplemented with 2 mM CaCl_2_ at 37°C.

### Whole-genome synthesis of bacteriophage S13 and its variants in the absence of natural templates

First, the whole-genome sequence of wild type bacteriophage S13 (GenBank accession number AF274751.1) to be synthesized was engineered by using custom-designed software, which accepted a DNA or protein sequence and inserted substitutions sites desired into the sequence. To generate m1-S13, we introduced two of the same changes (T to C) into the wt-S13 sequence at positions 1585 and 1777, which resulted in the creation of two *HaeIII* restriction sites. We also engineered two nonsynonymous mutation sites to generate m2-S13. The base and amino acid changes were as the following: nt 1301 (A to G; Thr to Ala); nts 1304 and 1305(T to G, C to G; Ser to Gly). We replaced the F capsid protein open reading frame of phage S13 with the F capsid protein ORF of phage G4 (GenBank accession number NC_001420.2) to produce a chimeric phage. Next, long genes were divided into short custom-made single stranded DNA oligonucleotides suitable for synthesis. All standard oligonucleotides were chemically synthesized and gel purified. The reason for purification was to reduce the error rate and keep oligo length limited. Then complementary oligonucleotides were annealed and recursively elongated with a heat-stable DNA polymerase to ultimately yield synthons≈1000 bp in length. At last, these synthons were efficiently assembled into the complete genomes of all four phages (wt-S13, m1-S13, m2-S13 and S13/G4 chimera) with low error frequency by fusion polymerase chain reaction (fusion PCR). The full-length DNAs were amplified by PCR with excess concentration of two gene-flanking primers to obtain a large number of accurate copies. All of these productions were completed and provided by Shanghai Sangon Biotech. Co., Ltd. A thorough description of the synthesis was presented in Material S1.

### Circularization of the linear full-length S13 DNA molecules

The S13 phage genomes were digested with restriction endonuclease *PstI* (Sangon Biotech Co., Ltd, Shanghai, China). The received linear genomes were gel purified and religated at a concentration of 1ug/ml with T4 DNA ligase (Sangon Biotech Co., Ltd, Shanghai, China). Then we precipitated the circular genomes with ethanol.

### Application of electroporation to deliver phage DNA into *E.coli* strain C

*E.coli* C cells were grown in liquid LB medium mixed with 10 mmol/L Mg2+ at 37°C until a cell density corresponding to OD600  = 0.6–0.8 was reached. 40ul of electro-competent cells was mixed with 1 ug of each phage DNA(wt-S13, m1-S13, m2-S13and S13/G4 genomic DNA chimera) and electroporated at 2100 V, 5 ms, in 0.2-cm cuvettes using a Eppendorf Electroporator 2510 (Sangon Biotech Co., Ltd, Shanghai, China). The electroporated cells were grown for 1h at 37°C after addition of 800μl of LB medium. Aliquots of the samples were then incubated overnight at 37°C on double-layer agar plates.

### Detection of engineered genetic markers in m1-S13 DNA

Single plaque was picked out for phage particles purification and amplification. Then bacteriophage DNA was isolated by using the method described previously [29]–[30]. Specific primers for wt-S13 and m1-S13 were synthesized by Sangon Biotech. (Shanghai), and their sequences were L,5′TTAATGCCACTCCTCTTC3′ and R,5′TCTTTAGTCGCAGTAGGC3′. The 628bp regions containing two given genetic markers in m1-S13 were generated and amplified during PCR, according to manufacturer's instructions. The conditions were as follows: 99°C for 2 min, followed by 30 cycles of 98°C, 10 s, 68°C, 40 s, 72°C, 10 min and a final extension of 72°C for 7 min. The 25 µl PCR mixture consisted of 1µl of phage DNA, 2.5 µl PCR buffer, 1.0 µl dNTP mix (100 mM each), 1 µl primer, 1.0 µl Taq DNA polymerase and 19.5 µl distilled water. The 5ul PCR products was either treated or untreated with *HaeIII* restriction endonuclease (Sangon Biotech Co., Ltd, Shanghai, China) at 37°C for 5 h. The resulting phage DNA fragments were separated on 2.0% agarose gels at 110V for about 50 minutes. Electrophoretic analysis of the results to determine the length of the DNA by comparing its electrophoretic mobility with a DNA marker sample (D2000marker).

### Phage DNA sequencing

A large plaque was picked from each plate directly into PCR mixtures for amplification. Sequences were obtained from PCR products of phage DNA as template, using a 3730 XL sequencer (3730 XL sequencer, ABI, Sangon).

### Phylogenetic analysis

Based on the DNA sequences of our synthetic phages and the sequences of other S13-related bacteriophages that gathered from GenBank (GenBank Accession Numbers: FJ849058.1,GQ153915.1,GQ153923.1,GU385905.1,J02482.1,AF454431.1,NC_001422.1,and NC_001420.2), we constructed of the phylogenetic tree by using maximum parsimony program with MEGA v 5.0 software. DNA sequence clusters between the synthetic S13-like phages and other previously sequenced microvirid phages were analysed.

### Study on the biological characteristics of the synthetic phages

Samples of the phage supernatant for wt-S13, m1-S13, m2-S13 and S13/G4 were collected from the four sequenced plaques.

Spectrophotometric monitoring of growth was performed to determine the killing effects of synthetic phages compared with the un-infected strain. Samples were taken at intervals of 30 min to measure the changes in OD600.

Titers of the four different phages were determined by the agar layer method after a 30-min exposure at 25, 35, 45, 55, 65, 70°C. pH stability test(pH values ranging from 3 to 10)was carried out by using the same method. Phage suspensions were exposured to UV light (20W, 50cm) and taken at intervals for plaque counts.

A method modified from that described by Bull JJ [25] was used to calculate adsorption rates. *E.coli* C was grown to a density of 1×10^8^/ml in LB medium supplemented with 2 mM CaCl_2_ in 125-ml asks at 37°C shaking at 200 rpm in an orbital water bath shaker. Approximately 106 phage were added to 10ml cultures of E.coli C. 100 ul of the mixture was plated immediately for determination of the titer of total phage (N0). A 1-ml sample was centrifuged at 12,000 g for 5 min at 4°C. The titer of unadsorbed phage (N_u_) in the supernatant was then determined. The formula N_u_ =  N_0_e^−kCt^ was used to estimate the adsorption rate (k was the adsorption rate, C the density of bacteria and t was measured in minutes). We then re-estimated the results from this study in LB medium without CaCl_2._

### Statistical analysis

Results were representative of three independent experiments. Student's unpaired two-sided t test was used for statistical analyses. SPSS software (version 17.0) was applied throughout and differences were considered statistically significant at P<0.05.

## Supporting Information

Material S1 **A detailed description of genome construction.**
(DOC)

File S1 **Phage DNA sequencing reports.**
(RAR)
