# Supplementary material for: Whole-Genome Synthesis and Characterization of Viable S13-Like Bacteriophages
Source: PLoS One. 2012 Jul 18;7(7):e41124. doi: 10.1371/journal.pone.0041124 (PMC3399791; doi:10.1371/journal.pone.0041124)
Supplement: Material S1 — A detailed description of genome construction. (DOC) [file pone.0041124.s001.doc]

**Supplemental Material**

**Protocol for assembly of synthetic oligonucleotides**

Based on the information from the supplier (Sangon Biotech Co., Ltd, Shanghai, China), we ascertained that 7 key steps were contained in the protocol: (1) All oligos to be synthesized were designed by using gene design software (http://software.kosan. com/GeMS). GC content, restriction enzyme sites and secondary structures were considered. The software then outputted a series of desired oligonucleotide sequences. (2)A large number of 40-nt oligos were chemically synthesized by using the phosphoramidite method. The overlapping region of complementary oligonucleotides was on average 20 nt long. (3) Gel purification of the oligos to eliminate undesirable errors. The recovered oligos were ethanol-precipitated and dissolved in double distilled water. (4) Purified 40-nt synthetic oligos were assembled into ‘synthons’ by using a polymerase cycling reaction. Briefly, equal volumes of the synthetic oligos were mixed together in a test tube and this mixture was added into a 25 µl reaction solution contains: 2.5µl of 10 × PCR buffer, 2.5µl of 10 × dNTP, 4µl of MgCl_2_, 1U of Taq polymerase. PCA parameters were 95°C for 30 sec, 52°C for 30 sec, and 74°C for 30 sec for 55 cycles, finishing with 74°C for 5 min. (5) These synthons were then progressively fused together into larger DNA fragments (each about 2700bp) in a series of low-cycle high fidelity ‘stitching’ long PCRs. The conditions for these PCR reactions were as follows: 10 cycles at 95°C for 20 sec, 70°C for 1 sec, 55°C for 30 sec, 68°C for 5 min; 5 cycles at 94°C for 20 sec, 70°C for 1 sec, 55°C for 30 sec, 68°C for 5 min; 10 cycles at 94°C for 20 sec, 70°C for 1 sec, 55°C for 30 sec, 68°C for 5-9min (increase extension time by 20 sec for every subsequent cycle). The final fused products were purified using the QIAquick Gel Extraction Kit (Qiagen, Valencia, CA, USA). (6) The resultant fragments were finally combined into the complete genomes in another PCR reaction for 12 cycles. (7) The full-length products were subjected to PCR amplification with two gene-flanking primers. The 50ul PCR mixtures contained 1ul of HF polymerase, 5ul of 10× pfu buffer, 1ul of dNTP, 1ul of DNA template and 1ul each of 10 uM primers. The PCR conditions were 28 cycles at 94°C for 18 sec, 55°C for 30 sec, 72°C for 90 sec and one cycle 72°C for 5 min.


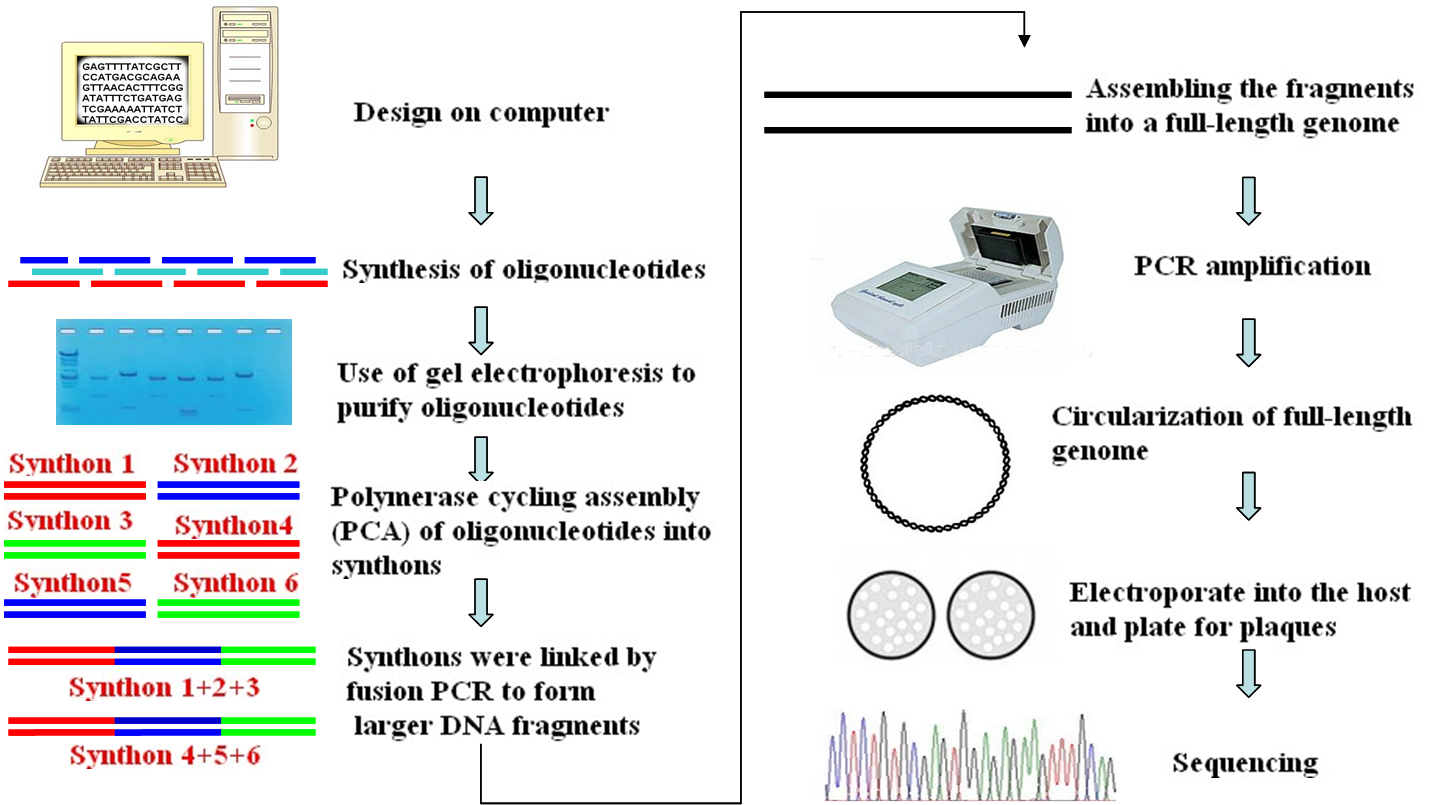


Fig.1 Schematic diagram of the steps in the chemical synthesis of viable bacteriophages from synthetic oligonucleotides.


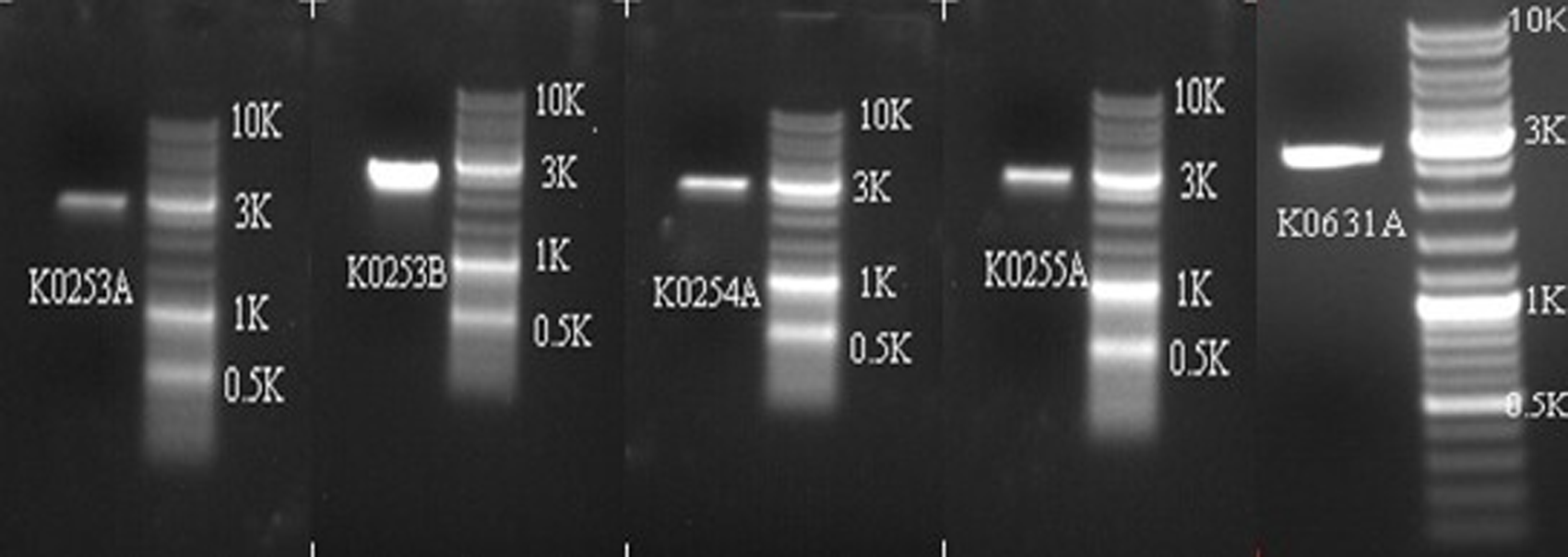


Fig. 2 Gel electrophoretic analyses of the fusion PCR products. K0254A, segment for wt-S13; K0253B, segment shared by each of our synthetic phages; K0254A, segment for m1-S13; K0255A, segment for m2-S13; K0631A, segment for S13/G4 chimera.
